# Obituary

**Published:** 1889-06

**Authors:** 


### OBITUARY.

DR. GEORGE WASHINGTON TUCKER was born at Pine Ridge, Miss., November 9, 1825. At the age of 9 years he was sent to Princeton, N. J., to be prepared for college. He finally entered Princeton College and graduated with honor at the age of 17. He remained at home for two years, when he went to Philadelphia, where he graduated in medicine at the University of Pennsylvania. He was married in 1852 to Miss Margeret Stuart Glover, and removed to Lafourche Parish, La., where he resided as a sugar planter, until the late civil war caused him to take his family to Texas for safety. During the war he was engaged in hospital work at Mansfield, La., and elsewhere.

Brought up a strict Presbyterian, five years after his marriage he became a member of the Protestant Episcopal Church, and was confirmed by the Right Rev. Leonidas Polk, Bishop of La., who was his dear friend and near neighbor for years. He was a member of the vestry and warden of St. John's church, Thibideaux, La., for many years. After his removal to New Orleans, in 1871, he was a member of the vestry of Emanuel church, which was afterwards united with St. Marks, forming the present parish of St. Georges, N. O.

He came to Texas in 1874, locating for six or eight months in Dallas, and then removing to Comanche, where he has since resided. In the epidemic of 1878 he went to Memphis and was put on the Howard corps of physicians, rendering faithful and honored services, as attested by the medal which was given him.

Always the genial, courteous gentleman, the well-read and conscientious physician, the faithful friend, the affectionate father, the humble, unostentatious disciple of his Master, his death, (which occurred after prolonged suffering, on Saturday, June 8,) will leave an aching void, which time alone can assuage.

He belonged to other and brighter days of our Sunny South. The following lines were found written on the fly-leaf of one of his note books, and expressed what we believe was his own faith as to the great change that has taken place with reference to himself.

"There is no death;
The stars go down to rise upon
Some fairer shore,
And bright in Heaven's jewelled crown
They shine forevermore.

Dr. Tucker was married, in 1881, to Mrs. Penn Tucker, who survives him, as well as two sons and three daughters by the first marriage. [One of the daughters is the wife of Rev. W. D. Sartwell, Rector of the Episcopal church, Corsicana.—Ed.]

---

Attention is called to two advertisements of practice for sale.

